# Supplementary material for: Are carbon emissions trading and green financial instruments synergistic? -Comprehensive quantitative research based on content analysis
Source: PLoS One. 2024 Mar 7;19(3):e0298601. doi: 10.1371/journal.pone.0298601 (PMC10919601; doi:10.1371/journal.pone.0298601)
Supplement: S1 Data — (ZIP) [file pone.0298601.s001.zip › Data/地级市Z_F工作报告.zip!/─■╧─/╩»╫∞╔╜╩╨/└┤╫╘╣1⁄2╓┌║┼í╛┬φ┐╦╩2╛▌═°í┐_2018.docx]

各位代表：

现在，我代表市人民政府，向大会报告工作，请予审议，并请市政协委员和列席人员提出意见。

一、2017年工作回顾

过去一年，在自治区党委、政府和市委的坚强领导下，在市人大和政协的监督支持下，市人民政府紧紧围绕打造石嘴山经济升级版、打响“山水园林·尚工逸城”城市品牌，大力实施“三个一”工程，迎难而上，主动作为，奋力攻坚，全市经济社会发展稳中有进、稳中提质。预计实现地区生产总值580亿元，增长7%；完成固定资产投资550亿元，增长8%左右；一般公共预算收入22.8亿元，可比增长4%；城乡居民人均可支配收入分别达到28050元、12840元，增长8%和8.5%。

（一）转型升级取得新成效。出台开发区转型升级三年行动计划、企业亩产效益综合评价办法等32项政策措施，打好转型升级组合拳。开展“双百”大会战、“无冬闲”活动，114个重点项目完成投资241亿元，重大产业、工业技改、高新技术产业投资分别增长11.5%、17%、21%，民间投资保持较好增长势头，投资结构不断优化。做好“浴火重生”“腾笼换鸟”文章，实施54个传统产业改造提升项目，兼并重组企业12家，处置“僵尸企业”41家，清理闲置土地7975亩，淘汰落后产能6.3万吨，退出煤炭产能525万吨；杉杉能源正极电池材料等项目建成投产，新材料、先进装备制造等新兴产业增速加快，预计规上工业增加值增长7%。实施企业成长培育计划，完成个转企58家、小升规19家，在“新三板”挂牌2家、区域性股交中心挂牌26家。农业供给侧结构性改革深入推进，国家农业科技园区通过验收，农作物制种等优势特色产业带动作用明显，农业生产保持稳定，预计增加值增长4.5%。“三争”工作成效明显，争取各类资金60.3亿元，增长19.3%；成功列入全国首批老工业城市和资源型城市产业转型升级示范区，制定出台三年行动计划纲要并加快推进落实。老工业基地调整改造工作得到国务院通报表扬。

（二）经济发展增添新动力。做好“无中生有”文章，新经济新业态新产业发展势头良好，预计服务业增加值增长8.5%。与阿里巴巴共建互联网城市，淘宝大学宁夏培训基地落户我市。网络经济产业园建成运营，入驻企业25家。出台支持金融产业发展“17条”，设立新光新经济产业投资基金，科技金融广场建成开业，入驻企业18家，实现交易额30亿元。与自治区食药监局共建大健康产业园，西北健康谷挂牌运营。大沙湖旅游区运营体制改革方案和开发规划得到自治区的肯定和支持，中华奇石山、北武当生态旅游区成功创建国家4A级景区，全市接待游客320万人次、旅游收入23亿元，分别增长12%和18%。文化创意产业园、黄河国际数字电影小镇、“煤城记忆·汉唐九街”特色街区建设加快推进。保税物流中心（B型）完成主体工程，曹妃甸港设立石嘴山内陆港，惠农陆路口岸发运各类货物592万吨，增长25%。

（三）创新能力实现新提升。实施科技创新“双倍增”三年行动计划，新增高新技术企业8家、科技型中小企业30家、国家级创新创业孵化器6家，万人有效发明专利拥有量达到4.5件，增长47%，增幅居全区第一，预计R&D 投入占GDP比重达到1.15%。建成石嘴山科技园，入驻科研平台及服务机构28家。出台支持创新创业“15条”，新增各类市场主体11400户、小微企业2610户。加大招才引智力度，引进高层次人才110名、科技创新团队4个。新申请注册商标270件，新创建农产品品牌4个，“惠农枸杞”成功注册国家地理标志证明商标。以改革促创新，开展“最多跑一次”改革，推行并联审批、网上审批、模拟审批，政府投资项目、企业投资项目办理时限分别压缩23个和29个工作日。完成空间规划（多规合一）改革试点，农村土地制度改革三项试点工作得到国家部委肯定，城市公立医院综合改革经验在全国推广。

（四）开放合作迈出新步伐。深入推进东西部合作交流，与杭州、金华、绍兴、苏州等城市签订友好合作协议，在产业合作、干部交流等方面达成20多项合作事项。与江浙企业和商会达成17项合作协议，计划投资170亿元。设立环渤海、长三角、珠三角3支专业招商队伍，选派13名干部到江浙地区挂职，学习先进理念。创新“三包三带”方式，开展精准招商，实际到位资金445亿元，增长10%。对外经济稳步扩大，预计全市实现进出口总额 35 亿元，增长56%。

（五）生态环境得到新改善。大力实施“三治”工程，整改完成中央环保督察反馈问题23个。以壮士断腕的决心打好贺兰山清理整治攻坚战，清理整治点118个，修复治理面积近5万亩，保护区内所有企业及保护区外围93家储煤场、洗煤厂关闭退出，贺兰山面貌明显改善。坚决打赢蓝天保卫战，淘汰燃煤锅炉377台，超低排放改造火电机组4台，淘汰小火电机组11台，空气质量优良天数达254天，增加18天。全面推行河长制，完成沙湖与星海湖水系连通工程，取缔入黄排污口7个，提标改造城镇污水处理厂6座，实施水污染防治项目39个，沙湖和星海湖水质由劣V类提高到V 类，黄河石嘴山段Ⅲ类水质达标率为100%。深入开展城乡环境综合整治，新增营造林面积2.7万亩、生态公园2个。

（六）城乡建设步入新阶段。扎实开展“六城联创”，举全市之力成功创建全国文明城市、国家卫生城市。启动“城市双修”十大工程，改造棚户区6303套、老旧住宅小区108万平方米，建设城市地下综合管廊4.7公里。出台房地产去库存、高校毕业生落户购房补贴政策，销售商品房74.4万平方米，房地产去库存成效明显，人口外流得到有效改善。启动特色田园小镇、田园乡村建设，加快推进新型城镇化，共同缔造美丽家园。红果子镇列入全国特色小镇，陶乐镇庙庙湖村列入全国美丽宜居村庄。

（七）民生事业收获新成果。持续加大民生投入力度，民生支出占财政支出的75%，十件民生实事全面完成。脱贫攻坚扎实推进，3009名建档立卡贫困人口实现脱贫，完成324户1590人易地搬迁安置任务。大力促进就业创业，新增城镇就业2.3万人，城镇登记失业率控制在3.8%。实施教育“三名”工程，基础教育各项指标均居全区前列，成立浙江师范大学石嘴山教师培训中心，宁夏理工学院与浙江工业大学开展合作办学，星海中学建成投入使用。实施卫生“三名”工程，互联网医联体上线运营，在全区率先开展家庭医生签约服务，公共卫生服务水平不断提高。全面放开养老服务市场，提前实现“十三五”每千名老人拥有机构养老床位目标。开展“广场文化艺术节”等演出465场次，承办第十五届中国戏剧节演出6场次，举办环星海湖绿色骑行节等系列赛事，丰富了群众文化体育生活。开展食用农产品市场准入试点活动，餐饮行业“4D”管理经验在全区推广。开展“三减”专项行动，启用智慧城市指挥中心暨“12345”便民服务中心，安全事故死亡人数、环保投诉、刑事发案、信访总量分别下降23.3%、13.9%、19.3%、12%。全面贯彻落实党的民族宗教政策，促进民族团结、宗教和顺。

（八）自身建设有了新提高。制定出台市人民政府进一步加强自身建设的决定，健全完善工作规则和议事制度，围绕市委决策部署狠抓贯彻落实，主动向市人大及其常委会报告工作，向市政协通报情况，办理市人大代表建议88件、政协提案181件。推进法治政府建设，大力推行政务公开，开展电视问政，解决了一批群众关心的热点、难点问题。加强审计监督，促进节支1.8亿元。坚持把纪律和规矩挺在前面，加大行政监察和整治“四风”力度，公开通报50起违反中央八项规定精神典型案例，严肃查处环境保护等重点工作中不作为、慢作为、乱作为等问题，问责处理111人，政风行风不断好转。

各位代表，一年来取得的成绩，是自治区党委、政府和市委坚强领导的结果，是市人大及其常委会、市政协大力支持的结果，是全市人民团结一心、拼搏奋斗的结果。在此，我代表市人民政府，向全市广大干部群众和离退休老同志，向市人大代表、政协委员、各民主党派、工商联、人民团体和各界人士，向驻石部队、武警官兵、消防官兵、驻石中央和区属单位，致以崇高的敬意！向关心支持石嘴山发展的朋友们，表示衷心的感谢!

在总结成绩的同时，必须清醒看到，我们的工作还存在许多不足，也面临不少困难和问题。主要是：发展不平衡不充分问题仍然突出，创新能力不足，传统产业层次不高，工业发展粗放，现代服务业弱；经济增长基础不牢固，大项目、好项目偏少，新的增长点支撑作用不足，财政收支矛盾突出；长期积累的生态环境问题正在集中显现，环境保护、污染治理和生态修复任务艰巨，节能降耗形势严峻；城市能级偏低，产城融合、协调发展不够，对人口的集聚和吸附能力不强；教育、医疗、文化、就业等公共服务水平，与群众日益增长的美好生活需要还有一定差距；一些部门和干部主动担当意识不强，改革开放意识不强，执行力不强，工作拖沓，效率不高。对此，我们一定要高度重视，下大力气解决。

二、2018年重点工作

2018年是贯彻党的十九大精神的开局之年，是改革开放40周年、自治区成立60周年，是决胜全面建成小康社会、实施“十三五”规划承上启下的关键一年，做好各项工作意义重大。我们要切实增强创新意识、担当意识、机遇意识、责任意识，贯彻新理念、聚焦新目标、落实新部署，推动质量变革、效率变革、动力变革，努力把短板变跳板、把挑战变机遇、把痛点变亮点，促进经济社会持续健康发展。

政府工作的总体要求是：深入学习贯彻党的十九大精神，以习近平新时代中国特色社会主义思想为指引，按照自治区党委、政府和市委的决策部署，贯彻新发展理念，牢牢把握高质量发展根本要求和稳中求进工作总基调，聚焦全面建成小康社会目标，以重大项目推进年、作风建设深化年活动为抓手，大力实施创新驱动、生态立市、脱贫富民“三大战略”，持续推动产业民生生态“三大转型”，着力打造石嘴山经济升级版，打响“山水园林·尚工逸城”城市品牌，提升人民群众的获得感幸福感安全感，建设新型工业之城、创新创业之城、生态美丽之城、和谐宜居之城，打造银川都市圈靓丽北翼，推动石嘴山由全区工业的“摇篮”向科技创新的“摇篮”转变、由工矿时代向生态时代转变，为决胜全面建成小康社会奠定坚实基础。

主要预期目标是：地区生产总值增长8%左右；固定资产投资增长8%；一般公共预算收入增长6%；社会消费品零售总额增长8.5%；城乡居民人均可支配收入分别增长8%和8.5%；完成自治区下达的节能降耗、污染减排、安全生产等约束性指标。

各位代表！实现上述目标，我们必须创新发展思路、发展方式和发展路径，努力做到“四个坚持”：

坚持发展实体经济不动摇。落实打造经济升级版系列政策，推进全国老工业城市和资源型城市产业转型升级示范区建设，加快传统产业改造提升，推动产业发展由价值链中低端向中高端转变、经济发展由资源开发型向效率提升型转变、增长方式由粗放型向集约型转变，建设新型工业之城。

坚持创新驱动战略不动摇。实现创新发展，企业是主体，产业是关键，根本是增强自主创新能力。以互联网城市建设为统揽，加快从要素驱动向创新驱动转变，走出一条机制创新、路径创新、模式创新的发展之路，建设创新创业之城。

坚持绿色发展理念不动摇。像对待生命一样对待生态环境，大力实施生态立市战略，整治好生态环境、修复好生态系统、发展好绿色经济，切实把生态修复转化成生态资源，把生态资源转化成生态资本，把生态资本转化成生态财富，共同缔造美丽家园，建设生态美丽之城。

坚持以人民为中心不动摇。始终把人民利益摆在至高无上的地位，实施脱贫富民战略和乡村振兴战略，切实保障和改善民生，带领人民共同创造美好生活，使人民获得感幸福感安全感更加充实、更有保障、更可持续，建设和谐宜居之城。

在具体工作中，我们必须创新工作方法，在“破”“立”“降”上下功夫，综合运用“加减乘除”法，加快实施“四轮驱动”：

实施投资拉动，做好“加法”。实现高质量发展，要落实在高质量的项目上、高质量的投资上。加大投资与需求相结合、与创新相结合，通过有效益的投资、有创新的投资、有质量的投资，拉长产业链条、培育产业集群、加快补齐短板，做大增量、做活存量、做优质量，“加”出新实力。

实施生态撬动，做好“减法”。实现高质量发展，要采取超常措施、拿出过硬办法，坚决打好污染防治攻坚战。通过生态环境容量控制，倒逼淘汰落后产能、倒逼企业降低能耗、倒逼提高资源效率，打破对资源、环境等传统要素的路径依赖，走绿色发展之路，“减”出新潜力。

实施创新驱动，做好“乘法”。实现高质量发展，要更加注重从创新中获取动力。把新观念、新科技、新要素、新举措作为支撑，突破发展新经济新业态新产业，带动产业结构优化、城乡结构优化、收入结构优化，释放创新带来的裂变效应，乘”出新动力。

实施改革推动，做好“除法”。实现高质量发展，要做好深化改革文章，激发各类市场主体活力。坚持导向思维、系统推进原则，充分发挥政府有形之手和市场无形之手的叠加效应，推动重点领域和关键环节改革取得新突破，实现制度体系、政策体系、工作体系的重新组合，去除制约和障碍，“除”出新活力。

要抓好九个方面重点工作：

（一）以项目为支撑，持续扩大有效投资。

强化“以项目论英雄、以项目论担当、以项目论能力”的工作导向，围绕“三资三流”，落实“五有”要求，提高项目质量和效益，提升投资有效性和精准性，切实增强发展后劲。

提速项目建设。坚持“资金围绕项目投、人才围绕项目引、政府围绕项目转”，开展“双百”大会战，完善市级领导“四联系”制度，建立健全项目引进—生成—前期—落地—建设“一条龙”协作服务机制，力促项目早开工建设、早投产达效。围绕工业转型升级，强力推进36个工业项目；围绕新经济新业态新产业发展，突出抓好26个服务业项目；围绕推动乡村振兴发展，提速建设11个农业项目；围绕城市能级提升，加快实施37个社会事业项目；围绕交通和环境改善，持续推动25个基础设施项目，确保135个重点项目完成投资230亿元以上。

狠抓招商引资。把招商引资作为“一号工程”，围绕“六个转变”，紧盯战略性新兴产业、新经济新业态，充分发挥三个驻外招商联络处、各地商会的作用，开展系列专业招商活动，以“蚂蝗精神”精准招商，着力招大引强、招高引新、招群引链。改进招商引资考核办法，对重大产业项目引进进行奖励，争取在具有战略意义的项目引进上取得重大突破，全年招商引资到位资金增长10%以上。

优化投资结构。投资的有效性主要取决于投资结构。更加重视投资结构调整、效率提升，着力推进“四提高、两下降”，重大产业、工业技改、高新技术、民间投资分别增长13%、15%、20%、10%以上，进一步降低房地产投资和政府投资比重。做好聚财、理财、生财工作，着力提高财政资金使用绩效。扎实开展“三清理”，清缴税费1亿元以上，清理闲置和低效用地5000亩以上，清理盘活国有资产。加大“三争”工作力度，争取资金增长15%以上。支持企业扩大直接融资，积极防范化解信贷风险，增强金融服务实体经济能力。

（二）以工业为根本，做大做强实体经济。

把实体经济作为“一号引擎”，突出工业主导地位，打造高端园区，培育高端企业，建设高端平台，推进产业高端化、智能化、集聚化发展，规上工业增加值增长8%以上。

发展智能制造。加大“互联网+”“机器人+”“标准化+”力度，大力推进“两化融合”，重点抓好100个工业技改项目，着力增链、延链、补链、强链。建立新材料、电石化工等产业技术联盟，对标对表改造提升冶金、煤机装备制造等传统产业，加快发展新材料、新能源、先进装备制造等新兴产业，新兴产业产值增长20%以上，占规上工业比重达到15%以上。

提升企业品质。大力支持民营经济发展，实施企业成长培育行动计划、企业上市和并购培育计划，深入推进“四换三名”，支持优势企业开展兼并重组，重点培育一批行业龙头企业和“隐形冠军”，新增产值30亿元以上企业2家、20亿元以上企业3家，完成个转企60家、小升规25家、规改股10家、“新三板”挂牌2家、区域性股交中心挂牌20家以上。力争创建中国驰名商标1个、宁夏名牌10个，推进质量强市和品牌强市建设。

提档升级园区。围绕“五化四链”目标，统筹推进产业差异化发展，高新区大力发展以新材料、先进装备制造为主的高新技术产业，培育发展大健康产业，加快追赶发展；经开区重点发展新能源和精细化工产业，培育发展轻质材料产业，加快换道发展；生态经济区推动特色冶金、电石化工产业循环发展，培育发展汽车零部件产业，加快绿色发展。切实抓好工业开发区转型升级三年行动计划落实，工业园区投资、产值和税收分别增长15%以上，推进产业集聚区建设。全力打好“四退四进”攻坚战，狠抓设施配套完善，加快推动工业园区向现代产业园区转型。

（三）以“三新”为引擎，再造转型发展优势。

实施现代服务业发展三年行动计划，提速发展新经济新业态新产业，加快培育形成新动能，服务业增加值增长9%以上。

大力发展全域旅游。加快大沙湖旅游区开发建设，推动沙湖星海湖板块联动发展，推进大武口、平罗全域旅游示范区创建。加快“煤城记忆·汉唐九街”特色街区建设，积极利用工矿遗址等资源，打造旅游新景区。 开展星海湖休闲度假区、中华奇石山、北武当生态旅游区组团创建5A级景区工作，新增4A级景区2家以上、3A级景区6家以上。坚持补短板、强弱项，加快推进“厕所革命”，大力发展观光游、休闲游、体验游、康养游、民宿游，延长旅游产业链，拥抱全民旅游时代。新增旅行社及分支机构10家以上，建设四星级酒店，升级住宿品牌，吸引游客在石平均停留时间2天以上，全市游客接待量、旅游收入分别增长15%和20%以上。

加快发展现代物流。提升惠农陆路口岸功能，深化与天津港、曹妃甸港及乌力吉口岸等合作，促进公路、铁路、水路多式联运，吸引更多货物从我市集港并中转。高标准建设保税物流中心，创新发展服务外包产业，发挥“口岸+保税”的独特优势，突破发展保税物流、跨境电商物流。推进惠农富海冷链物流、大武口快递物流园区、国际物流园平罗站改扩建等项目，建设现代物流产业集聚区，现代物流业增加值增长10%以上。

突破发展新兴业态。加快数字经济的深度融合，推进网络经济产业园扩容提质，新增企业40家以上，实现网上交易额15亿元以上。大力发展电子商务，培育综合性电商龙头企业和行业性电商平台10家以上。实施科技金融发展三年行动计划，科技金融广场新增企业20家以上，实现年交易额80亿元以上。加快文化创意产业园、黄河国际数字电影小镇建设，开发体验性影视游演艺节目，创建名人工作室20家以上，引进线上线下影视制作关联企业100家以上。推进划行归市，培育专业街区、专业市场，打造辐射蒙西地区的商业核心区块。积极培育养老、健康等消费热点，西北健康谷新引进企业15家以上，构建大健康产业体系。

（四）以“三转”为路径，推进乡村振兴发展。

大力实施乡村振兴战略和脱贫富民战略，加快农业转型、促进农村转美、推动农民转行，推进农业农村现代化。

加快农业转型发展。深入推进农业供给侧结构性改革，大力发展绿色种植、生态养殖等高效生态农业，建设一二三产融合发展现代农业园区3个，将国家农业科技园区建成现代农业改革创新示范区，让单一的传统园区农业经济向“眼球经济”拓展，着力补齐农业短板。大力发展“互联网+农业”，实施农产品品牌提升计划，健全农产品质量追溯体系，提升农产品生产、加工、流通、营销服务水平，打造现代农业全产业链，新增市级以上农业产业化龙头企业10家、特色农产品品牌10个，新培育示范性合作社10家、家庭农场20家，促进农业产业化稳步发展，力争农业增加值增长4.5%以上。

加快田园乡村建设。实施共同缔造美丽家园试点行动计划，启动建设田园综合体，加快推进村庄整合、人居建设、环境整治、产业增收和乡风文明培育，集中力量打造10个特色田园乡村。努力实现试点村供水普及率、饮用水达标率、互联网宽带入户率、垃圾处理率、污水处理和改厕入户率“五个100%”，村庄绿化覆盖率达到35%，建成新农村建设的样板村。开展共同缔造美丽家园“十佳村”“十差村”评选活动，推动特色田园乡村从“一处美”向“一片美”转型。

加快脱贫富民步伐。坚持脱贫为先、富民为要，深入实施“四项脱贫计划”和“五个助力行动”，注重扶贫同扶志、扶智相结合，突出产业扶贫、科技扶贫、金融扶贫，加大贫困群众就业技能培训力度，激发贫困人口内生脱贫动力，以项目带动贫困村发展，确保年度建档立卡贫困人口脱贫。大力实施富民工程，落实好强农惠农富农政策，挖掘农业生产经营性收入，增加务工工资性收入，释放财产性收入红利，拓展农民收入增长空间。加快推进乡村旅游全域化、农村电商全域化，培训新型职业农民600人以上，推动农民转行、转产。通过发现培育一批、吸引回乡一批、外地招引一批，培育农村致富带头人50人以上，带动更多群众增收致富。

（五）以创新为引领，增强全新发展动力。

加快实施科技创新“双倍增”行动计划和中科协创新驱动助力工程，积极推进沿黄科技创新改革试验区建设，充分发挥科技创新在全面创新中的引领作用。

突出创新平台建设。石嘴山科技园新增各类创新主体20家以上，年内创建成为自治区级科技孵化器。支持企业组建技术协同创新中心和创新战略联盟，在高新区、经开区、生态经济区建立科创中心，助推产业转型发展。扩展全国小微企业双创示范基地建设成果，新增众创空间、科技孵化器5家以上，培育科技型初创企业50家以上，新增市场主体1万户以上，打造“双创”升级版。

推进科技成果转化。加大中小企业创新支持力度，设立科技创新基金，鼓励企业增加研发投入，新增自治区级及以上研发中心、技术创新中心5家，新增高新技术企业8家、科技型中小企业30家，推进产学研一体化，促进科技成果转化。加快推进100个科技合作项目建设，科技进步贡献率达到52%以上，R&D 投入占GDP比重达到1.5%左右。

加快创新人才集聚。全面落实人才政策，引进一批科技领军人才、青年科技人才和高水平创新团队，培养一批具有工匠精神的高技能人才，造就一支具有“四千”精神的企业家队伍。全年引进创新创业人才200名以上，柔性引进区外专家50名以上；培训企业经营管理人才、专业技术人才、农村实用人才2万人次以上，鼓励回乡创业，努力形成产业高地、人才洼地。

（六）以活力为重点，不断深化改革开放。

推出一批叫得响、立得住、群众认可的改革实招，提供更加精准有效的制度供给，扩大东西部合作交流成果，吸引更多更高端的资源要素汇聚。

加快审批制度改革。大力推广“互联网+政务服务”，扩大“多证合一”覆盖范围，推行淘宝式全流程网上审批办理、移动端APP应用，打通各部门信息数据通道，让数据多跑路，让群众少跑腿。推行不见面审批服务改革，建立重大项目审批“绿色通道”，构建网上办、集中批、联合审、区域评、代办制、不见面的审批模式，网上在线审批达到100%，不见面办理事项达到80%，政府投资项目、企业投资项目办理时限分别控制在80个、60个工作日以内。

深化重点领域改革。深化投融资体制改革，创新财政投入方式，组建市属国有资本投资运营公司，做大做强国有投融资平台，政银企合作设立产业基金。深化市属国有企业改革，发展混合所有制经济，健全现代企业制度，激发发展活力，提高市场竞争力。深化生态文明体制改革，实施环保执法扁平化管理，促进生态文明建设。深化园区管理体制改革，完善市、辖区、园区行政审批体制，建立园区投融资平台，增强园区发展内生动力。深化农村综合改革，稳步推进集体产权制度改革，统筹推进“三权分置”，重点抓好平罗县改革试点扩面提升，把“盆景”变成“风景”，把“风景”变成“全景”。

扩大对外合作交流。围绕发展开放型经济、建设开放型城市，以“学习新经验、扩大朋友圈、招引好项目”为目标，深化东西部合作交流，扩大与杭州、苏州等城市的合作成果，加快产业、项目、技术、人才交流合作。举办与深圳、宁波、无锡、镇江等城市的合作交流活动，既注重资金、资本、项目的引进，也注重发展理念、发展模式、高端人才的引进。办好呼包银榆经济区第六届联席会议，构筑优势互补、良性互动的区域发展新格局。大力培育外向型经济，加快传统对外贸易向现代国际贸易转型，积极引进外资，实现进出口总额增长50%以上。

（七）以品质为关键，全面提升城市能级。

聚焦提升品质和补齐短板，加快推进新型城镇化、城乡一体化，巩固提升全国文明城市创建成果，着力打造银川都市圈靓丽北翼，建设“三生三宜”城市。

推进银川都市圈建设。坚持城市规划同城化、产业发展差异化、交通设施一体化、公共服务均等化，加快形成特色化。大武口区要加快发展，打造高新技术产业示范区、新经济新业态新产业集聚区、生活性服务业核心区；惠农区要转型发展，打造工业转型升级示范区、文化创意产业试验区、生产性物流集聚区；平罗县要科学发展，打造现代工业示范县、科学发展样板县、西部中等发达县。以建设产业、生态、旅游“三条廊道”为抓手，加强与宁东装备制造、能源化工等产业协作，共同参与要素配置、产业分工和市场竞争，形成大武口—惠农—平罗产业集聚区；加快推进包银高铁石嘴山段等交通基础设施建设，推动构建交通“一张网”，形成一小时经济圈、生活圈、旅游圈；以石银跨界区域水环境整治为重点，实施艾依河整治、贺兰山生态治理工程，加强生态环境共治。深化都市圈城市文化教育、社保医疗、社会治理、政务服务等领域合作，推动公共服务对接共享。

增强城市承载能力。积极推进空间规划（多规合一），修编新一轮城市总体规划，提高城市设计水平，优化城市空间布局。实施城区街景及亮化改造、特色街区塑造等工程，争取列入国家“城市双修”推广城市。加强城市精细化管理，继续加大棚户区改造力度，推进“三供一业”家属区改造，完善新区功能配套，做精老城区、做优新城区。落实房地产去库存10条政策，完善教育、医疗、住房、社保等9项配套政策，推进农业转移人口市民化，留住本地人、吸引外地人，新增外来人口5%以上。加强公共交通基础设施建设，积极稳妥推进三县区城市公交一体化。加快互联网城市建设，启动大数据和 5G 网络建设，大力推行公交“一卡通”和供水、供电、供暖缴费移动支付，让群众享受移动互联网带来的便利。

打造特色田园小镇。坚持“一镇一业”“一镇一品”“一镇一韵”，按照“四高一低”的总体要求，科学编制沙湖古镇、绿色产业小镇、特色美食小镇等10个特色田园小镇规划。推进产业、文化、旅游深度融合，谋划实施一批支撑性产业项目、一批基础设施项目、一批社会事业项目、一批文化传承项目，特色田园小镇完成投资20亿元以上，其中产业投资不低于60%，初步完成创建的基础性工作。推行“小镇+景区”形态模式，所有特色田园小镇按照3A级景区标准规划建设，其中旅游产业类小镇按照4A级景区标准规划建设，推进镇区景区化、景区全域化。

（八）以绿色为导向，加快生态文明建设。

牢固树立“绿水青山就是金山银山”的理念，深入开展“蓝天碧水·绿色城乡”专项行动，构建高标准环境保护综合治理体系，还百姓天蓝、地绿、山青、水净的生态环境，让山水园林、绿色生态成为石嘴山最大的财富、最大的优势、最响的品牌。

整治生态环境。打好“三治”组合拳，找准污染源，切断污染源，根治污染源，持续推进中央环保督察反馈问题整改。深化贺兰山清理整治工作，切实把父亲山的生态环境整治好、修复好、保护好。打好新一轮蓝天保卫战，开展“四尘”同治，严格执行环保政策和相关行业标准，狠抓小、散、乱、污企业清理整治，空气质量优良天数比例达到73%以上。严格落实河长制， “一河一策”推进72条河湖治理，加强水源地保护，重点实施第三第五排水沟治理、艾依河石嘴山段人工湿地等项目，沙湖、星海湖水质稳定在Ⅴ类，黄河石嘴山段水质达到或优于Ⅲ类比例稳定在100%。大力开展农业面源和畜禽养殖污染治理，加强固体废弃物处置能力建设，推进生活垃圾资源化、减量化、无害化处理。

修复生态系统。统筹山水林田湖草系统治理，巩固国家森林城市、国家园林城市创建成果，推动国家生态文明先行示范区建设。开展“百万花灌木进城”活动，增加街头绿地、小微游园，丰富城市色彩，全面消除城区裸露土地。大力实施绿色通道、工业园区绿化、乡村美化等重点生态项目，加快推进110国道生态廊道、星海湖生态修复、平罗“五湖四海”生态公园、惠农城南生态景观提升等工程建设，打造城市景观水体，建设滨水生态长廊，实现“湖在城中、城在园中” “房在绿中、绿在景中”，构筑“山水环润”的生态新格局。

发展绿色经济。坚决不要发臭的GDP，积极推动传统产业绿色化转型、重点企业清洁化生产、工业园区生态化改造，构建绿色产业链和资源循环链。出台工业园区产业准入负面清单，提高环保准入门槛，实行环保评价“一票否决”。运用企业亩产综合效益评价结果，按投资强度、经济密度、税收贡献率，以环保、节能、安全等约束性指标，对“低产田”和“高产田”进行差别化要素配置，倒逼落后产能淘汰退出和低效企业转型转产。按照要素资源差别化配置，列出名单、拿出计划、加快推进，处置“僵尸企业”40 家以上，促进资源高效利用。倡导绿色生活方式，扎实推进生态园区、生态镇、生态村创建工作。

（九）以人民为中心，着力增进民生福祉。

坚持在发展中保障和改善民生，既尽力而为，又量力而行，解决好群众最关心最直接最现实的利益问题，不断满足人民日益增长的美好生活需要，实现经济发展与民生改善的良性循环。

把准民需之脉。优先发展教育事业，坚持立德树人，大力实施教育“三名”工程，组建教育集团，启动市域义务教育基本均衡发展试点工作，加快发展学前教育，提升职业教育办学水平，加强与浙江、上海等发达地区院校的学科领域合作，全面提升教育教学质量。大力实施卫生“三名”工程，儿童医院和妇幼保健院建成投入使用，推进综合医改和公立医院改革，深化医联体建设，打造一流学科，扩大家庭医生签约服务范围，促进优质医疗资源“双下沉、两提升”。实施文化名人文艺精品工程，重点扶持七大领域24部优秀作品，开展文艺作品下基层演出500场次以上，丰富群众精神文化生活。加快市民健身公园建设，举办全国高水平体育赛事，做好自治区第十五届运动会备战工作，广泛开展全民健身活动，推进“健康石嘴山”建设。

织密保障之网。实施积极的就业政策，解决好高校毕业生、就业困难人员等重点群体的就业问题，新增城镇就业2万人，城镇登记失业率控制在4%以内，努力增加城镇居民收入。推进全民参保计划，健全城乡统筹和区域转接机制，稳步提高保障水平。加快构建和谐劳动关系，全面治理拖欠职工、农民工工资问题。做好全国居家和社区养老服务改革试点工作，宁夏银北养老院建成运营，增强养老服务供给能力。完善社会救助体系，努力实现困难群众应救尽救、应养尽养。大力发展残疾人事业、社会福利和慈善事业，保障妇女儿童、青少年、老年人合法权益。

铸牢安全之盾。深化“平安石嘴山”建设，大力实施“雪亮工程”，加大公共安全设施投入，构建以网格化管理为基础的社区治理体系，创建全国社会治安综合治理优秀城市。扎实开展“七五”普法，增强全民法治意识，争创全国法治城市。加强食品药品监管，守护群众“舌尖上的安全”，争创国家食品安全示范城市。深入开展民族团结进步创建活动，提升宗教事务法治化管理水平，争创全国民族团结进步示范市。加快社会信用体系建设，提高城市综合信用指数。充分发挥“12345”服务热线作用，打通群众诉求的“最后一公里”。 着力解决群众合理诉求，积极化解信访案件。加大“三减”工作力度，确保安全事故死亡人数下降5%以上，环保问题投诉下降15%以上，信访总量下降12%以上。

各位代表，今年我们将按照“群众提、政府办”的要求，切实办好十件民生实事。同时，继续支持工会、共青团、妇联等群团组织工作，重视做好人口计生、军民融合、双拥共建、防震减灾、统计调查等工作。

三、努力建设人民满意政府

各位代表，做好经济社会发展各项工作，对政府工作提出了新要求。我们要以更高标准、更严要求、更实作风，加强政府自身建设，全面提升政府的执行力、战斗力和公信力。

(一）依法行政，建设法治政府。我们一定要创新法治思维，依法作为。把法治理念贯穿政府工作全过程，做到授予有据、行使有规、施政有信、监督有效。健全重大行政决策程序，科学决策、民主决策、依法决策。主动接受人大及其常委会依法监督、政协民主监督，自觉接受社会监督和舆论监督，加强行政复议和行政应诉工作，促进依法行政。高度重视并认真办理人大代表建议意见和政协提案。加强诚信政府建设，全面推进政务公开，让权力在阳光下运行。

（二）高效施政，建设服务政府。我们一定要创新工作方法，主动作为。资源枯竭但作风不能枯竭，要减少“二传手”、争当“主攻手”，减少“裁判员”、争当“运动员”，切实改变“履责”变“推责”的现象，以钉钉子精神做实做细做好各项工作，推动事业和产业共发展。牢记宗旨观念，做好“五送”，当好“五员”，争当“店小二”，把群众的小事当作政府的大事，马上就办、马上办好，多干让群众有获得感的实事，多干雪中送炭的好事，多干百姓急需解决的难事，用政府的“不自在”换取群众的“更自在”，用我们的辛苦指数提升群众的幸福指数。

（三）实干勤政，建设责任政府。我们一定要创新履职方式，担当作为。幸福都是奋斗出来的，一分耕耘，一分收获。要坚持说实话、谋实事、出实招、求实效，把精力和心思集中到研究问题、分析问题、解决问题上来，把工作当作事业干、把事业当作使命干，抓紧每一天、干好每件事，对标先进，抓特色、争一流、树亮点。建立重点工作清单制度，明确推进时间表、路线图、施工图，做到有督查、有考核、有问责，确保“政策不睡觉、落地见实效”。健全完善议大事、报难事、查要事工作机制，推行扁平化管理，践行“一线工作法”，承诺的事情坚决兑现，既定的事情坚决办成。

（四）从严治政，建设清廉政府。我们一定要创新监督机制，廉洁作为。牢固树立“四个意识”，严守政治纪律和政治规矩，巩固拓展落实中央八项规定精神成果，驰而不息纠正“四风”。完善廉政风险防控机制，严肃查处违法违纪案件，坚决整治侵害群众利益的不正之风和腐败问题。建立常态长效的追责问责机制，严厉整肃庸政懒政怠政，坚决整治不作为、慢作为、乱作为。严格财经纪律，严控“三公”经费，把每一分钱都花在刀刃上。作风建设永远在路上，各级领导干部要带头转变作风，身体力行、以上率下，用政府的“紧日子”换取群众和企业的“好日子”。

各位代表，新时代开启新征程，新时代赋予新使命。让我们更加紧密地团结在以习近平同志为核心的党中央周围，在自治区党委、政府和市委的坚强领导下，锐意进取、埋头苦干，焕发新状态、展现新作为、创造新业绩、交出新答卷，为决胜全面建成小康社会、实现人民对美好生活的向往而努力奋斗!
